# Dynamics of anti-RBD (anti-receptor binding domain) levels in diabetes patients following the ChAdOx1 nCoV-19 vaccine (AZD1222) in the Thai population

**DOI:** 10.1038/s41598-023-39114-5

**Published:** 2023-07-21

**Authors:** Supamas Sirisreetreerux, Thachanun Porntharukchareon, Bothamai Dechates, Vimonsri Rangsrisaeneepitak, Phonthip Therawit, Supanat Worawitchawong, Gaidganok Sornsamdang, Kamonwan Soonklang, Kriangkrai Tawinprai

**Affiliations:** 1 Department of Medicine, Chulabhorn Hospital, Chulabhorn Royal Academy, 906 Thung Song Hong, Lak Si, Bangkok, 10210 Thailand; 2 Central Laboratory Center, Chulabhorn Hospital, Chulabhorn Royal Academy, 906 Thung Song Hong, Lak Si, Bangkok, 10210 Thailand; 3 Center of Learning and Research in Celebration of HRH Princess Chulabhorn 60th Birthday Anniversary, Chulabhorn Royal Academy, 906 Thung Song Hong, Lak Si, Bangkok, 10210 Thailand; 4 Division of Endocrine and Metabolism, Department of Medicine, Burapha Hospital, Saen Suk, Mueang Chonburi, 20131 Thailand

**Keywords:** Immunology, Endocrinology

## Abstract

The ChAdOx1 nCoV-19 vaccine (AZD1222) was used in Thailand during the early outbreak of coronavirus disease 2019 (COVID-19). A previous study showed a low immune response in diabetes patients after the first dose of the AZD1222 vaccine. Furthermore, humoral immune responses after the second vaccination were inconsistent. This study evaluated the immunogenicity following the first and second doses of the AZD1222 vaccine in people with type 2 diabetes (T2D) compared with the general population of Thailand. This was a prospective, single-center cohort study. 59 adults with T2D and 118 age- and sex-matched healthcare personnel were eligible. The participants received two doses of AZD1222 12 weeks apart. Antibodies against the receptor-binding domain (anti-RBD) of the SARS-CoV-2 spike protein, using an automated electrochemiluminesence immunoassay (ECLIA), were measured at baseline, 8 and 12 weeks after the first dose of vaccine, and 4 weeks after the second dose of vaccine. The anti-RBD levels were reported as the geometric mean concentration (GMC) and compared between groups using the geometric mean ratio (GMR). A total of 177 participants were included: The average age of 59 T2D patients was 60.1 years (SD: 11.4), and 31 (52.5%) of them were female. The GMC of anti-RBD 8 and 12 weeks after the first vaccination were significantly lower in T2D (week 8 60; 17.05 BAU/mL, 95% confidence interval [CI]   11.1–26.19, *P = *0.035, week 12; 24.68 BAU/mL, 95% CI  16.4–37.0, *P = *0.002) than in those without diabetes (week 8; 29.79 BAU/mL, 95% CI  22.07–40.42, week 12; 50.67 BAU/mL, 95% CI  40.62–63.20). However, there was no difference in the GMC of anti-RBD 4 weeks after the second vaccination among groups (T2D; 687.95 BAU/mL, 95% CI  462.7–1022.7, Normal; 697.95 BAU/mL, 95% CI 583.7–834.5, *P = *0.947). In both groups, the GMC of anti-RBD was persistently high without decline 12 weeks after the first vaccination. Albuminuria was a major factor related to low humoral immune responses in T2D patients after the second dose of AZD122 vaccine (the GMR was 0.29, 95% CI 0.08–0.98, *P = *0.047) whereas the HbA1C level and age were not. Immunogenicity in T2D cases was lower than in the normal population after the first dose of the AZD1222 vaccine. The two doses of AZD122 vaccine induced immunity in T2D equal to that of normal individuals in Thailand. People with diabetes should be boosted as soon as possible to induce adequate immunity to prevent COVID-19 infection.

## Introduction

SARS-CoV-2, a novel coronavirus that causes coronavirus disease 2019 (COVID-19) was first reported in China in December 2019. The World Health Organization (WHO) declared a COVID-19 pandemic in March 2020^1^. Diabetes mellitus is considered a high-risk factor for poor outcomes of COVID-19 infection. People with diabetes were associated with a twofold greater risk of mortality than the general populations^2^. Moreover, diabetic patients were more likely to be hospitalized and require admission to an intensive care unit^3,4^. Therefore, COVID-19 vaccination is highly recommended for diabetes patients because of their poor adverse COVID-19 outcomes. Most policies worldwide, including Thailand, prioritized vaccination for people with diabetes; however, during the early outbreak, the quantity and type of COVID-19 vaccines were limited. In first phase, there are two vaccine strategies in Thailand. The CoronaVac vaccine was administered as two doses 2–4 weeks apart and the ChAdOx1 nCoV-19 vaccine (AZD1222) was administered as two doses 8–12 weeks apart^5^.

The ChAdOx1 nCoV-19 vaccine (AZD1222) is one of the COVID-19 vaccines used in Thailand. It was approved by Thailand’s Food and Drug Administration (FDA) for emergency use in January 2021 and indicated for the active immunization of individuals aged 18 years and older. It consists of the replication-deficient chimpanzee adenoviral vector expressing the full-length SAR-CoV-2 spike protein. The WHO recommends two doses given intramuscularly (0.5 ml each) with an interval of 8 to 12 weeks^5^. In the general population, the AZD1222 vaccine was 76.0% (95% CI   59.3–85.9) effective against symptomatic COVID-19 infection from day 22 to 90 days after a single standard dose, and 63.1% (95%CI   51.8–71.7) effective at 14 days after two standard doses. The immunogenicity after the second dose was twofold higher in those with a homologous prime-boost interval of 12 weeks or more compared with those with an interval less than 6 weeks. (GMR 2.32, 95% CI  2.01–2.68)^6^ Therefore, when there was a low supply of vaccine, a strategy was proposed to delay the second dose of vaccine to allow a greater proportion of the population to be vaccinated. In Thailand, the government recommended receiving the second dosage of the AZD1222 vaccine 12 weeks after receiving the first dose due to a shortage of the vaccine in country. This period was of concern for high-risk populations.

Chronic dysmetabolic and inflammation in diabetes patients lead to accelerated immune aging^7^. Immunogenicity in diabetes patients was reduced after hepatitis B vaccination and less consistent results have been reported for influenza and pneumococcus vaccines^8^. During early outbreak, Thailand had the most SARS-CoV-2 Alpha (B1.1.7) variant outbreak (74.08%) together with the increased of the Delta (B.1.617.2) variant (24.14%) which high transmissibility, mortality, and morbidity rate. The emergence of variants of concern (VOC) was associated with decreased vaccine efficacy. Therefore, the concern arose regarding the immunological response in individual with diabetes following the AZD1222 vaccine. The vaccine efficacy in diabetic patients was 43.2% (95% CI 26.0–56.3) at 28–90 days after one dose of the AZD1222 vaccine and increased to 72.9% (95%  CI 25.8–90.1) at 14–69 days after the second vaccination^9^. A previous study reported a low immune response in diabetes patients after the first dose of the AZD122 vaccine and the older age was associated with weaker immune response^10^. However, after the second dose of COVID-19 vaccine, the immune response was similar between diabetes patients and the general population^11^. Most cases in that study were vaccinated with the BNT162b2 mRNA vaccine (86%). In this study, we used the AZD122 vaccine, the most commonly administered vaccine in the early outbreak of SARS-CoV-2 in Thailand.

This study evaluated the immunogenicity following the first and second doses of the ChAdOx1 nCoV-19 (AZD1222) vaccine in people with type 2 diabetes (T2D) compared with the general population in Thailand.

## Methods

### Study design and participants

This prospective study evaluated humoral immune responses after the first and second doses of the AZD1222 vaccine in people with T2D between June and October 2021. The advertisements on poster was performed. Patients with T2D who visited Chulabhorn Hospital were invited to participate in the study. We included adults with type 2 diabetes, aged 18 years and older who were willing to be vaccinated and who signed written informed consent. Healthcare personnel in our institute were enrolled as age- (within 5 years) and sex-matched controls. The diagnosis and classification of diabetes was made according to American Diabetes Association criterias^12^. Demographic data, comorbidities, diabetes complications, body mass index, glomerular filtration rate, albuminuria (defined as a urine albumin: creatinine ratio ≥ 30 mg/g), insulin use, and the most recent glycated hemoglobin (HbA1C) levels (the last 6 months) were collected from medical records.

Exclusion criteria were participants with a history of COVID-19 infection which confirmed by the serum of SARS-CoV-2 antibody level at baseline, those who received other COVID-19 vaccines during the study, pregnancy, breast feeding, and history of blood transfusion in the last 3 months. We also collected data on serious adverse events post-vaccination.

### Vaccination procedure

Participants received the AZD1222 vaccine with a dosing interval of 12 weeks. An intramuscular injection of 0.5 ml of vaccine into the deltoid muscle was administered to each individual. A venous blood sample was collected on day 0 before the first vaccination, and then 56 ± 7 days (8 weeks) and 84 ± 7 days (12 weeks) after the first dose, and 28 ± 7 days after the second dose (16 weeks after the first dose). Anti-RBD immunoglobulin were measured using the Elecsys Anti-SARS-CoV-2 S (Elecsys-S) kit (Roche Diagnostics, Mannheim, Germany), an automated electrochemiluminescence immunoassay (ECLIA). The measurement and validation were performed according to the manufacturer's instruction. The Elecsys-S uses the double-antigen sandwich principle to detect anti-S protein antibodies. Participants were observed in the clinic for 30 min after the vaccination procedure and asked to record any adverse events by using a mobile application on days 1 and 7 after the first and second vaccination.

### Study outcomes

The primary outcome was the geometric mean concentration (GMC) of anti-RBD antibodies after the second vaccination in people with and without type 2 diabetes 4 weeks after the second dose (T3). The secondary outcomes were the GMC of anti-RBD antibodies at 8 weeks (T1) and 12 weeks (T2) after the first vaccination in the diabetic group compared with healthy controls, dynamic immune responses after the first and second vaccination in the diabetic group compared with baseline, and factors associated with immunogenicity in the diabetic group.

### Sample size calculation

The sample size was calculated by testing two independent means (two-tailed test) based on a previous study^13^. People with diabetes were matched for age and sex at a 1:2 ratio with healthy controls. The estimated sample size was 169 participants (57 in the diabetic group and 112 in the control group) to achieve a power of 80% with a significance level of 5% (alpha = 0.05). This calculation was based on a participant drop out of 20%.

### Statistical analysis

Data were analyzed using STATA/SE version 16.1. Categorical variables were summarized as frequencies and percentages (%). Quantitative variables were summarized as means and standard deviations (± SD). The anti-RBD antibody concentration was summarized as the GMC and 95% confidence interval (CI). The GMC was compared between groups using the geometric mean ratio (GMR). Factors associated with GMC in the diabetic group were identified using univariate and multivariate analyses. The immunogenicity was compared between the groups using a multiple linear regression model. Adverse events related to the vaccine were analyzed by Pearson’s chi-square and Fisher’s exact tests. A *P*-value < 0.05 indicated statistical significance.

### Ethical approval and consent to participate

The authors declare that the research was conducted with no conflict of interest. The study protocol, case records form, and consent form were reviewed and approved by the Ethics Committee for Human Research of Chulabhorn Research Institute (056/2564) on June 7, 2021. This trial was registered with thaiclinicaltrials.org (TCTR20211228003), and the protocol conformed to the principles of the Declaration of Helsinki.

## Results

Overall, 59 patients with T2D and 118 age- and sex-matched healthcare personnel were recruited in this study. Demographic data are shown in Table 1. In the diabetic group, of 59 subjects, 28 (47.5%) were male and 31 (52.5%) were female, with a mean age of 60.2 years (SD: 11.4) of which 69% were at least 60 years old. The mean HbA1C was 7.43% (± 1.39) and 50% of cases had a level lower than 7%. The mean BMI was 26.44 (± 3.56) and 64.44% of cases were considered obese. Insulin usage was observed in 12 patients (20.34%). Diabetic retinopathy was found in 18 subjects (30.51%) and chronic kidney disease (defined as a glomerular filtration rate [GFR] calculated by the CKD-EPI formula ≤ 60 ml/min/1.73 m^2^) and albuminuria (defined as a urine albumin:creatinine ratio ≥ 30 mg/g) were found in 30 (49.1%) and 15 (25.42%) subjects, respectively. Most subjects had a comorbidity—44 subjects had hypertension (74.58%) and 56 subjects had dyslipidemia (94.92%).

**Table 1:** Demographic data of participants with type 2 diabetes and age- and sex-matched controls.

	Diabetes	Healthy controls
Sex n (%)		
Male	28 (47.46)	56 (47.46)
Female	31 (52.54)	62 (52.54)
Age (years), mean(SD)	60.19 (11.44)	59.52 (11.66)
Weight (kg), mean(SD)		
BMI (kg/m^2^), mean(SD)	26.44 ± 3.56	NA
HbA1C (%), mean(SD)	7.43 (1.39)	-
GFR (ml/min/kg/m^2^), mean(SD)	72.53 (26.51)	NA
LDL cholesterol, mean(SD)	92.92 (35.38)	NA
Insulin used, n(%)		
Non-used	47 (79.66)	–
Used	12 (20.34)	–
Diabetic retinopathy, n(%)	18 (30.51)	–
Albuminuria*, n(%)	15 (25.42)	–
Cardiovascular disease, n(%)	11 (18.6)	3 (2.54)
Hypertension, n(%)	44 (74.58)	19 (16.10)
Dyslipidemia, n(%)	56 (94.92)	16 (13.56)

BMI,  body mass index; GFR, glomerular filtration; HbA1c,  glycated hemoglobin; SD,  standard deviation.

*Albuminuria was defined as a urine albumin: creatinine ratio ≥ 30 mg/g.

**Figure 1:**
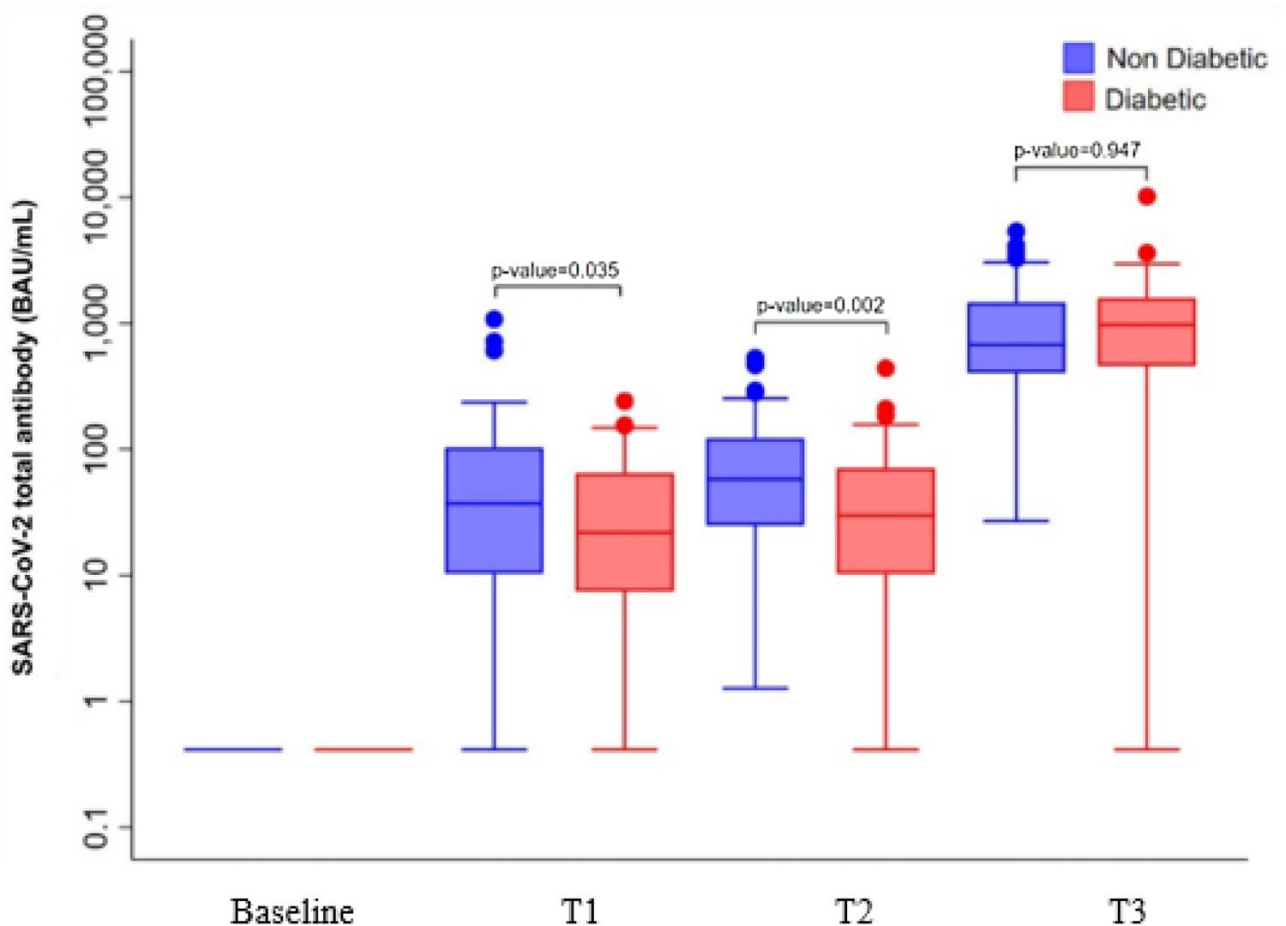
Mean antibody levels at 8 (T1) and 12 weeks (T2) after the first vaccination and 4 weeks after the second vaccination (T3) with the AZD1222 vaccine in diabetic patients and healthy controls.

The GMC of anti-RBD antibodies was significantly lower in diabetes patients than in healthy controls after the first vaccination at 8 weeks (T1) (17.05 BAU/mL, 95% CI  11.10–26.19 vs 29.79 BAU/mL, 95% CI  22.07–40.20; *P = *0.035) and 12 weeks (T2) (24.68 BAU/mL, 95% CI  16.43–37.09 vs 50.67 BAU/mL, 95% CI  40.62–60.20; *P = *0.002).The GMC of anti-RBD antibodies was not significantly (*P = *0.947) different between participants with diabetes (687.95 BAU/mL, 95% CI  462.75–1022.74) and healthy controls (697.95 BAU/mL, 95% CI 583.70–834.57) after the second dose of AZD1222 vaccine (T3) (Table 2, Fig. 1) and the GMR was 0.99 (95% CI  0.64–1.51).

**Table 2:** Mean antibody levels at 8 (T1) and 12 weeks (T2) after the first vaccination and 4 weeks after the second vaccination (T3) with the AZD1222 vaccine in diabetic patients and healthy controls.

	Antibody level (BAU/ml) geometric mean (95%CI)	Geometric ratio (95% CI)	*P*-value
8 weeks after first vaccination (T1)			
Healthy control	29.79 (22.07–40.20)	Ref. 0.57 (3.34, 0.96)	0.035
Diabetes	17.05 (11.10–26.19)		
12 weeks after first vaccination (T2)			
Healthy control	50.67 (40.62–63.20)	Ref. 0.49 (0.31,0.77)	0.002
Diabetes	24.68 (16.43–37.09)		
4 weeks after second vaccination (T3)			
Healthy control	697.95 (583.70,834.57)	Ref. 0.99 (0.64,1.51)	0.947
Diabetes	687.95 (462.75,1022.74)

A significant increase in anti-RBD antibodies was found after the first dose (T1 and T2) and second dose of vaccine (T3) compared with baseline in both groups. In the diabetic group, the GMC of anti-RBD antibody levels at T2 (24.68 BAU/mL, 95% CI 16.43–37.09) tended to be higher than at T1 (17.05 BAU/mL, 95% CI  11.10–26.19) after the first vaccination but this did not reach statistical significance (*P = *0.212). A similar trend in anti-RBD antibody levels at T2 compared with T1 was also found in healthy controls.

In the diabetic group, a significantly lower immune response was observed in participants with albuminuria compared with those without albuminuria. The GMC ratio was 0.29 (95% CI  0.08–0.98, *P = *0.047). Age, sex, obesity, HbA1c level, insulin use, GFR, and presence of diabetic retinopathy and cardiovascular disease did not significantly affect antibody levels (Table 3).

**Table 3:** Univariate and multivariate analyses of variables associated with GMC in individuals with type 2 diabetes.

	Antibody level (BAU/ml) (geometric mean (95% CI))	Univariate analysis		Multivariate analysis	
		Geomatic Ratio (95% CI)	*P*-value	Geomatic Ratio (95% CI)	*P*-value
Sex					
Male	686.27 (381.81, 1233.48)	Ref 1.00 (0.45, 2.24)	0.991		
Female	689.48 (389.77, 1219.65)				
Age					
< 65 years	618.68 (342.62, 1117.18)	Ref. 1.31 (0.63, 2.76)	0.465		
≥ 65 years	812.27 (505.50, 1305.20)				
HbA1C					
< 7.0	675.72 (398.07, 1147.04)	Ref. 1.04 (0.46, 2.31)	0.928		
≥ 7.0	700.84 (374.47, 1311.64)				
BMI					
< 23	1015.36 (641.98, 1605.92)	Ref. 0.64 (0.35, 1.15)	0.130		
≥ 23	647.20 (410.68, 1019.95)				
GFR					
< 60	644.89 (366.82, 1133.74)	Ref. 1.10 (0.52, 2.36)	0.798		
≥ 60	711.14 (413.26, 1223.72)				
Insulin use					
No	704.30 (432.36, 1147.28)	Ref. 0.89 (0.46, 1.73)	0.730		
Yes	627.48 (376.50, 1045.77)				
Cardiovascular disease					
No	703.21 (491.24, 1006.66)	Ref. 0.89 (0.20, 4.01)	0.876		
Yes	625.12 (116.69, 3348.97)				
Diabetic retinopathy					
No	715.09 (483.07, 1058.55)	Ref. 0.88 (0.31, 2.49)	0.808		
Yes	629.92 (225.89, 1756.63)				
Albuminuria					
No	955.90 (720.97, 1267.37)	Ref. 0.27 (0.08, 0.94)	0.041	Ref. 0.29 (0.08, 0.98)	0.047
Yes	262.13 (70.61, 973.15)				
Hypertension					
No	1159.79 (847.32, 1587.49)	Ref 0.50 (0.27, 0.90)	0.021	Ref 0.69 (0.40, 1.20)	0.184
Yes	575.75 (343.36, 965.42)				
Dyslipidemia					
No	1526.48 (819.93, 2841.89)	Ref. 0.43 (0.27, 0.70)	0.001	Ref. 0.52 (0.26, 1.03)	0.060
Yes	659.20 (435.25, 998.37)				

HbA1c = glycated hemoglobin, BMI = body mass index, GFR = glomerular filtration.

*Albuminuria defined as urine albumin/creatinine ratio ≥ 30 mg/g.

### Side effects of vaccination with AZD1222

Adverse events within the first day and 2–7 days after the second vaccination are presented in Table 4. Injection site reactions were more common in patients with T2D than in healthy controls within the first day (4 (6.77%%) vs 1 (0.85%), *P = *0.043). Participants with diabetes had more headaches and myalgia 2–7 days after the second vaccination compared with healthy controls. There were no differences between groups for other side effects including fever, fatigue, nausea, vomiting, diarrhea, and rash.

**Table 4:** Adverse events after two doses of AZD1222 vaccine in patients with type 2 diabetes and healthy controls.

Reaction	Day 1			Day 2–7		
	T2D patients (%)	Healthy controls (%)	*P* value	T2D patients (%)	Healthy controls (%)	*P* value
Injection site reaction	4 (6.77)	1 (0.85)	0.043	2 (3.38)	0 (0.00)	0.110
Fever	1 (1.69)	2 (1.69)	1.000	1 (1.69)	0 (0.00)	0.333
Headache	4 (6.78)	1 (0.85)	0.059	3 (5.07)	0 (0.00)	0.036
Fatigue	3 (5.08)	1 (0.85)	0.109	2 (2.38)	0 (0.00)	0.110
Myalgia	2 (2.38)	0 (0.00)	0.110	3 (5.08)	0 (0.00)	0.036
Nausea	1 (1.69)	1 (0.85)	0.557	2 (3.39)	0 (0.00)	0.110
Vomiting	0 (0.00)	0 (0.00)	1.000	0 (0.00)	0 (0.00)	1.000
Diarrhea	0 (0.00)	1 (0.85)	1.000	2 (2.38)	0 (0.00)	1.000
Rash	0 (0.00)	1 (0.85)	1.000	0 (0.00)	0 (0.00)	1.000
Other	0 (0.00)	0 (0.00)	1.000	1 (1.69)	0 (0.00)	0.333

## Discussion

This study demonstrated that people with T2D and healthy controls had similar humoral immune responses at 4 weeks (T3) after a second dose of the AZD1222 vaccine, as assessed by measuring the GMC of anti-RBD levels. In the diabetic group, albuminuria was associated with a weaker immune response after the booster dose, whereas age and HbA1C levels are not. Compared with the healthy controls, diabetes patients did not develop sufficient humoral immunity after the first dose of the AZD1222 vaccine; however, the GMC of anti-RBD levels at 12 weeks (T2) tended to be higher than those at 4 weeks (T1) although this did not reach statistical significance. These results suggest that humoral immune responses remained high and persisted for almost 12 weeks after the first dose of the AZD1222 vaccine in diabetes patients.

The previous study was carried out in England during the Alpha-variant dominant phase. Data was gathered from 7,480,272 registered patients at 718 English general clinics. the antibody levels from 28 to 90 days following the first dose of the AZD1222 vaccine were lower in the diabetic group (17.3 AU/mL, IQR 5.8–51.7) than in the non-diabetic group (30.6 AU/mL, IQR 11.1–63.5) and the GMR was 0.71 (95% CI 0.54–0.93). However, following the second dose of the AZD1222 vaccination, the immune response was comparable between the diabetic group (714 AU/mL, IQR 333-1580.5) and the non-diabetic group (774.5 AU/mL, IQR 364-1533), with a GMR of 1.02 (95% CI  0.85–1.23)^9^. These findings were consistent with our research. A homologous prime-booster of AZD1222 vaccine provided similar immunity in people with and without diabetes. A low immune response was observed after the first dose of the AZD1222 vaccine; therefore, people with T2D should be prioritized for an early booster vaccination with AZD1222, given their adequate immune response.

The findings of our study were in contrast to those of a homologous prime-booster of BNT162b2 mRNA vaccine, which induced similar immune responses in individuals with and without T2D since after the first (21 days) (T2D: 220.10 AU/mL, 95% CI  122.59–395.17 vs. normal: 354.62 AU/mL, 95% CI 268.34–468.65, *P = *0.144) and second (7–15 days) vaccinations. (T2D: 5300.64 AU/mL, 95% CI  3868.71–7262.56 vs. normal: 6281.32 AU/mL, 95% CI 5244.47–7523.16, *P = *0.350)^14^.

According to the Com-COV trial, heterologous prime-boost AZ/Pfizer (1387 ELU/mL, 95% CI   1186–1623) had a higher immunogenicity than homologous prime-boost AZ/AZ (1,2995 ELU/mL, 11,520–14,660) after 28 days of boost dosage and the GMR was 93 (95% CI 7.7–11.4). These two cohorts' participants, AZ/AZ and AZ/Pfizer, had diabetes in 7(8%) and 8(9%), respectively^15^. Although the heterologous prime-boost AZ/Pfizer may induce a greater immune response in diabetes patients than AZ/AZ, a future study specifically targeting diabetic patients may offer more reliable proof of an immune response.

A single dose of the AZD122 vaccine provided persistent immunogenicity for at least three months in the general population^16^. In our study, we also found this characteristic in diabetes patients. The antibody levels at 12 weeks (T3) remained high although they were lower than those in the healthy controls. This might be explained by the immunization of recombinant adenovirus exhibiting prolonged transgene expression resulting in the persistence of T-cell responses^17^.

Our study showed that poor glycemic control and older age did not correlate with lower antibody levels after a homologous prime-boost of AZD1222 vaccine. These results are consistent with a recently published single-blind, randomized, controlled, phase 2/3 trial (COV002 study)^18^, which reported a lower anti-RBD level with an increase in age after the first vaccination (group: 18–55 years; median 9807 AU/mL, IQR 5847–17,220, 56–69 years; 5496 AU/mL, IQR 2548–12,061 and ≥ 70 years; 4156 AU/mL, IQR 2122–12,595, *P = *0·0044) but with a similar immune response across three age groups 28 days after the second vaccination. (groups: 18–55 years; median 20,713 AU/mL, IQR 13,898–33,550, 56–69 years; 16,170 AU/mL, IQR 10,233–40,353 and ≥ 70 years; 17,561 AU/mL, IQR 9705–37,796, *P = *0·68). The COVAC-DM cohort study^10^ reported similar results to our study where HbA1C levels were not a predictor of immunity in T2D patients with HbA1C < 53 mmol/ml (7%) and T2D with HbA1C ≥ 53 mmol/ml after the second COVID-19 vaccination. Another study of the first dose of the AZD122 vaccine demonstrated older age and poor glycemic control were factors related to lower antibody levels^10^. These studies suggest that age and HbA1C do not affect immunogenicity after a homologous prime-boost of AZD122.

The CAVEAT study reported a significantly lower antibody response after a second COVID-19 vaccination in people with T2D with HbA1c > 53 mmol/ml (7%) compared with T2D patients with HbA1C < 53 mmol/ml (7%), which was in contrast to our study^19^. A reason for these different results might be that most participants with T2D in our study had well controlled diabetes (HbA1C < 7%), which may be related to the lack of statistical significance. Second, HbA1c levels were not measured at the time of vaccination in our study.

Albuminuria in our study was a major factor related to lower humoral immune responses in diabetic patients after the second dose of the AZD122 vaccine. An increase in albuminuria (urine albumin/creatinine ratio ≥ 30 mg/g) and decrease in GFR (≤ 60 ml/min/1.73 m^2^) are markers of chronic kidney disease (CKD). Antibody production by B lymphocytes was decreased by antigen presenting cell dysfunction, memory T cell apoptosis, and B cell lymphopenia, which are also present in CKD patients^20^, and might explain the lower immunity in T2D patients with albuminuria. In our study, a low GFR did not cause a low immune response. Further studies with a focus on people with diabetic kidney disease may provide more accurate data on vaccine efficacy in these cases.

Our study had some strengths. First, we studied patients with T2D who received only the AZD122 vaccine, which was the most common vaccine used during the early outbreak of SARS-CoV-2 infection in Thailand. Second, we measured antibody levels at different time points; therefore, we could clearly demonstrate interval changes of immunity in people with T2D after the first and second doses of the AZD122 vaccine. This study had some limitations. First, we used anti-RBD antibodies as a marker of vaccine efficacy instead of neutralizing antibodies, which are more predictive of immune protection from symptomatic SARS-CoV-2 infection^21^. Second, the study was conducted in a single center and the results may not apply to general populations. Finally, the study did not follow long-term immunity levels after the second vaccination; therefore, we were unable to determine the optimal duration for re-vaccination.

## Conclusions

Prime-boosting vaccination with AZD122 provided similar humoral immune responses in people with T2D and healthy controls in Thailand. The immune levels remained high and persisted for at least 3 months after the first dose of AZD122 in T2D cases although at levels lower than those in the normal population. These findings indicate that people with T2D should be boosted early to provide adequate immunity to prevent COVID-19 infection.

## Data Availability

The datasets used and/or analysed during current study available from the corresponding author on reasonable request.

## Abbreviations

AZD122: ChAdOx1 nCoV-19
T2D: Type 2 diabetes
anti-RBD: Antibodies against the receptor-binding domain
GMC: Geometric mean ratio
